# Surgical treatment of tracheal stenosis during Covid-19 era: a single-center experience and lessons learnt on the field

**DOI:** 10.1007/s13304-023-01577-6

**Published:** 2023-07-17

**Authors:** Diana Bacchin, Vittorio Aprile, Alessandra Lenzini, Stylianos Korasidis, Maria Giovanna Mastromarino, Alessandro Picchi, Olivia Fanucchi, Alessandro Ribechini, Marcello Carlo Ambrogi, Marco Lucchi

**Affiliations:** 1 Department of Surgical, Medical and Molecular Pathology and Critical Care Medicine, University of Pisa, Pisa, Italy; 2 Division of Thoracic Surgery, Cardiac, Thoracic and Vascular Department, University Hospital of Pisa, Pisa, Italy; 3 Thoracic Endoscopy Unit, Thoracic and Vascular Department, University Hospital of Pisa, Pisa, Italy

**Keywords:** Tracheal stenosis, Tracheal surgery, Covid-19, SARS-CoV2 pandemic

## Abstract

SARS-CoV2 outbreak led to several healthcare system challenges, especially concerning respiratory support to Covid-19 patients. In the first 2 years of pandemic (2020–2021), we assisted to a marked increase of post-invasive mechanical ventilation (IMV) tracheal stenosis incidence, many of them requiring surgical treatment, namely resection and primary end-to-end anastomosis. Our aim is to describe our experience during the abovementioned biennium, focusing on postoperative outcomes of patients who have had Covid-19 (“post-Covid”). We retrospectively collected pre-, intra- and postoperative data on all patients who underwent tracheal surgery for benign stenosis in our Unit from May 2020 to October 2021, including previous Covid-19. Comparison between “post-Covid-19” and “non-Covid-19” groups’ outcomes was then performed. Patients were 9 males and 6 females, and mean age was 57.4 ± 13.21 years. Nine patients had previous Covid-19 (60%). All patients underwent multidisciplinary preoperative evaluation. Almost every operation was conducted by cervicotomy, and the mean length of the resected specimen was 23.9 ± 6.5 mm. Postoperative complications were: dysphonia (3 cases), wound infection (1), bleeding (1), ab-ingestis pneumonia (1), anastomosis dehiscence (2), and stenosis recurrence (1). Thirty-day mortality rate was 6.7% (1 patient). No significant differences between “post-Covid-19” and “non-Covid-19” groups were identified. Pathology revealed, in 5 “post-Covid-19” patients, signs of vasculitis or perivascular inflammatory infiltrate. Tracheal stenosis’ surgical treatment has always been known as a challenging procedure because of high perioperative morbidity and mortality rates. Our experience shows that previous Covid-19 and ongoing pandemic did not significantly affect perioperative outcomes of patients who underwent tracheal resection and primary end-to-end anastomosis in a high-volume Center and after multidisciplinary workup.

**Graphical abstract:**

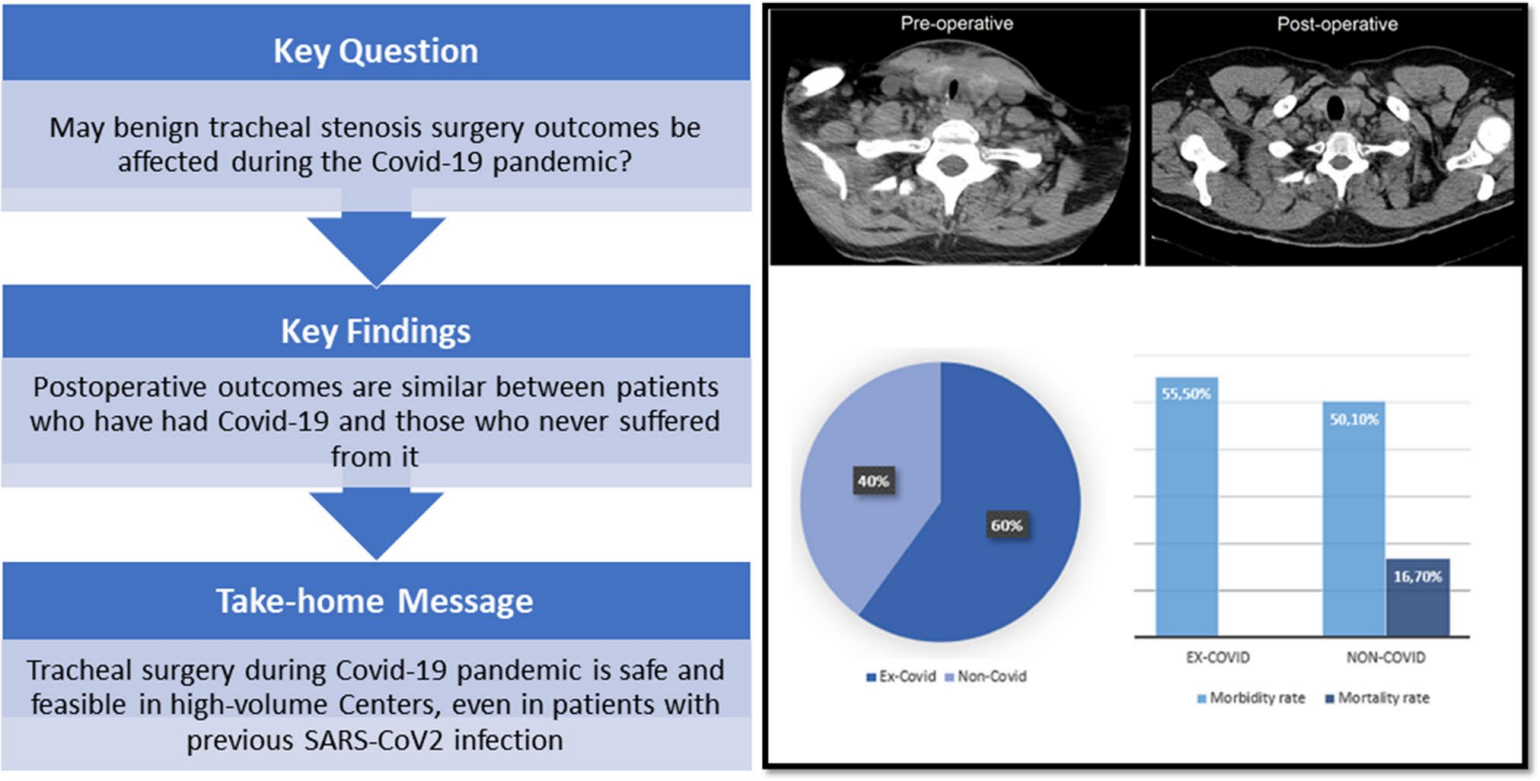

## Introduction

Tracheal stenosis is a potentially life-threatening condition and a major therapeutic challenge. Prolonged (> 14 days) invasive mechanical ventilation (IMV), namely trans-laryngeal intubation and tracheostomy, is the main cause of acquired laryngo-tracheal stenosis, occurring up to 20% of cases [1–3], due to the ischemic injury of the tracheal mucosa with subsequent circumferential scarring and narrowing of the involved area, caused by the pressure of the cuff on the tracheal wall [4]. Other causes could be researched in tracheo-esophageal fistula, traumatic airways injuries, autoimmune or autoinflammatory diseases, infections, malignancies, previous neck or mediastinum radiation therapy and congenital abnormalities.

Clinical presentation is usually characterized by acute or chronic dyspnea, accompanied by wheezing, stridor and retention of secretions, while other secondary symptoms may include cough, hemoptysis, and recurrent infection of the lower respiratory tract [5, 6].

Approximately, 5–12% of patients affected by Coronavirus disease 2019 (Covid-19) in Europe have been admitted to intensive care units (ICU) and up to 90% of them underwent prolonged IMV. As reported by Richards-Belle and colleagues in October 2020, Covid-19 patients admitted to ICU needed longer ventilatory support than patients affected by other viral pneumonias, so they were more likely to be tracheostomized [7].

Reports show that up to 5% of mechanically ventilated Covid-19 patients later presented chronic upper airways symptoms or some degree of tracheal stenosis [8].

As a result, from the beginning of the new Coronavirus (SARS-CoV2) pandemic, our Center witnessed a marked increase in the incidence of post-IMV tracheal stenosis [9], and many requiring surgical treatment usually after the failure of conservative endoscopic treatment.

## Methods

### Patients

Clinical, demographic, intra- and postoperative data on patients who underwent surgery for benign (laryngo)tracheal stenosis during the SARS-CoV2 pandemic (from May 2020 to October 2021) in our Center have been retrospectively collected. We included in the analysis patients who developed Covid-19-related respiratory failure requiring intubation and those who never experienced SARS-CoV2 infection. Whenever indicated by stenosis features and patient clinical status, a primary endoscopic treatment was performed (namely, laser disobstruction, balloon dilation, and stent positioning).

We decided to exclude from this analysis those patients who underwent tracheal resection for tracheo-esophageal fistula because of the additional complexity of surgical procedure and postoperative care related to this condition, whose description goes beyond the aims of our study.

Our primary endpoint was to analyze surgical short- and long-term outcomes of patients who underwent tracheal surgery (resection and primary end-to-end anastomosis) during the Covid-19 pandemic; secondarily, we compared surgical results of patients who experienced severe Covid-19 requiring IMV (“post-Covid-19”) and patients who did not (“non-Covid-19”).

This is an observational study. Every patient signed an informed consent to the use of their clinical data for research purposes. The University Hospital of Pisa’s Research Ethics Committee has confirmed that no ethical approval is required.

### Preoperative assessment

Every patient has been first evaluated in a multidisciplinary setting composed by a thoracic surgeon, a bronchial endoscopist, an anesthesiologist and a pneumologist. All patients underwent fiberoptic bronchoscopy (FOB) in order to evaluate the stenosis features, namely: histological diagnosis if neoplasm was suspected, morphology, distance from vocal cords, length of extension, residual tracheal lumen, dynamic characteristics, and cartilaginous rings involvement.

**Fig. 1:**
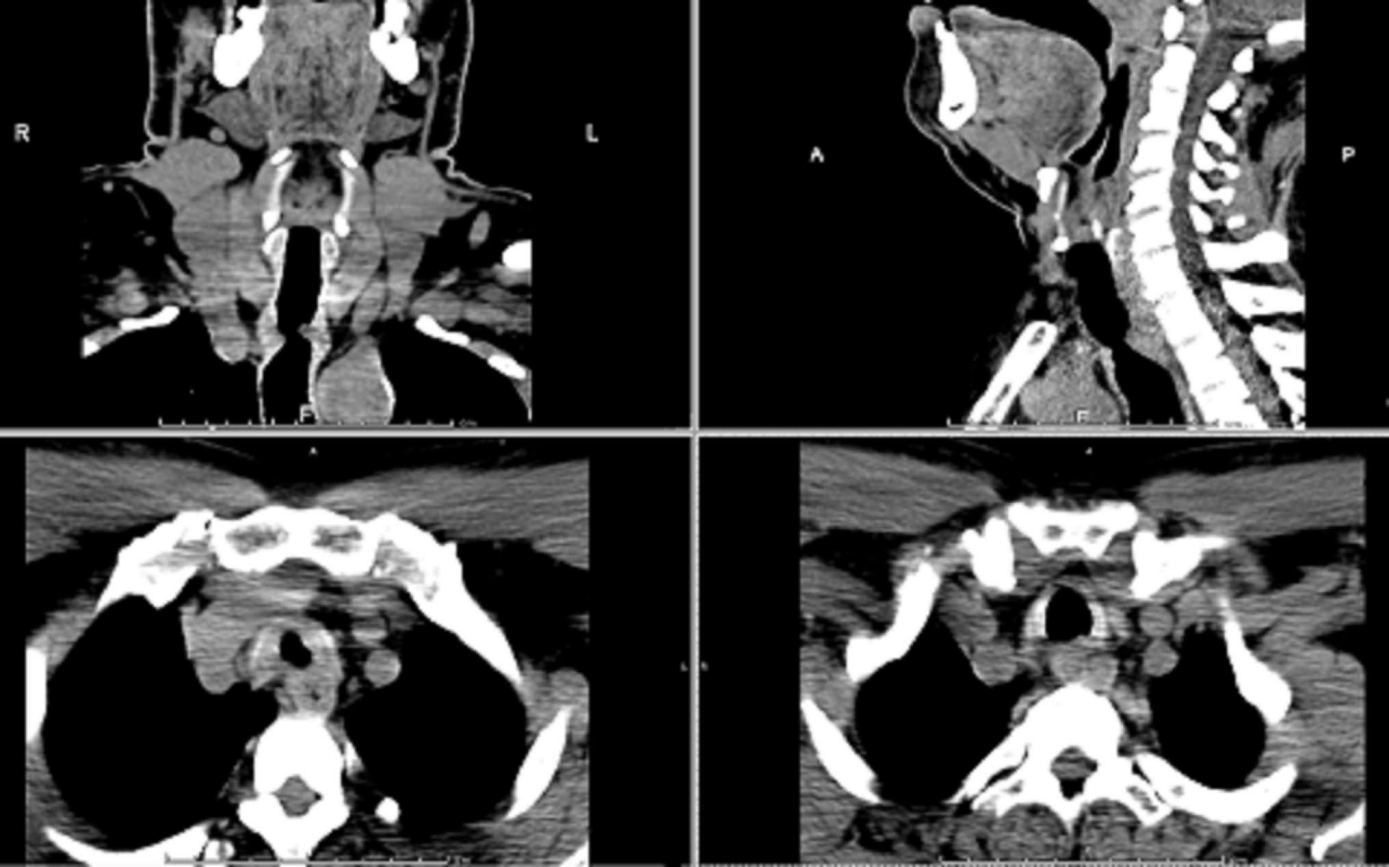
CT scan with multiplanar reconstructions of a patient affected by tracheal benign stenosis

In the same way, all patients underwent a neck and thorax computed tomography (CT) scan with multiplanar reconstructions (Fig. 1) to better assess the anatomical relationships of the tracheal stenosis with the surrounding organs.

At the moment of surgery, all post-Covid-19 patients were clinically healed from SARS-CoV2 infection and no viral RNA was detected at real time-PCR test performed on nasopharyngeal swab.

We did not set a precise timing between the SARS-CoV2 infection/ICU discharge and surgery, since surgical fitness of each patient was established at the moment of presentation. However, when possible, we preferred to wait at least 2 months after the acute event that required IMV to let patients heal from any eventual sequelae.

### Surgical procedure

The procedure, already described in a previous article [10], could be so summarized: under general anesthesia and, orotracheal intubation, all patients had an intraoperative FOB to confirm stenosis features and to support the anesthesiologist during intubation maneuvers. A transversal cervicotomy was carried out in all cases.

**Fig. 2:**
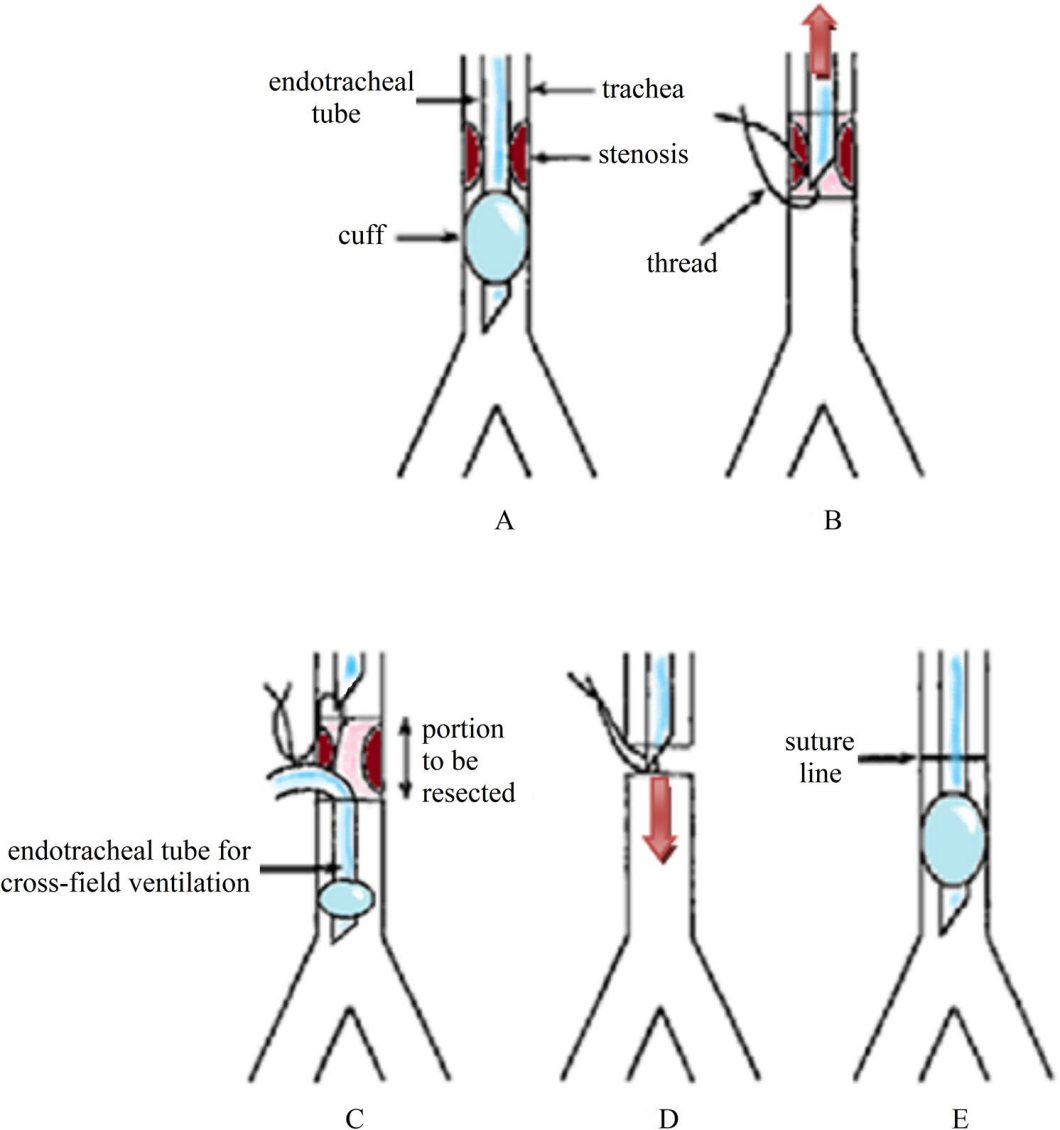
**A** Intubation, after dilatation of the stenosis; **B** marking of the orotracheal tube (OTT) with a thread after the trachea has been opened; C withdrawing of the OTT, insertion of an endotracheal tube (ETT) for cross-field ventilation and resection of the stenosis; **D** removal of the ETT and drawing back of the OTT tube just before completion of the anastomosis and placement of the cuff distally to the suture line; **E** completion of the anastomosis

Usually, we first resect the distal margin of the stenotic portion, detected by direct bronchoscopic view; then, after having marked the tip of the orotracheal tube (OTT) with a stitch, the tube is retracted without passing through the vocal cords (Fig. 2). An armored endotracheal tube (ETT) is then inserted in the distal stump and connected to the respiratory circuit. The superior margin of the stenosis is then identified and the affected tracheal rings were resected. The specimen is sent to the pathologists for examination. The primary end-to-end anastomosis is carried out using synthetic monofilament absorbable sutures, by continuous suture size 3-0 for the membranous wall and by separated stitches size 2-0 for the cartilaginous part.

Once the anterior stitches are applied, the ETT is removed, and the OTT is slipped down again. The anastomosis is then completed and tested for air leak. A silicone Redon drainage was placed in the pre-tracheal space before closing the surgical access. One or two chest-to-chin stitches in non-absorbable, braided thread are placed to avoid neck extension.

At the end of surgery, the patient is awakened and a FOB control is performed to assess the patency of the anastomosis.

### Postoperative management

After surgery, every patient was immediately extubated and FOB was carried out approximately in third and seventh postoperative days, or when needed in case of suspicion of anastomosis complications.

We usually avoid administering chronic steroid therapy, even if short-term, high-dosage corticosteroids are exceptionally required because of development of laryngeal edema immediately after surgery.

The chest-to-chin stitch and the neck drainage was removed after 7–10 days postoperatively if no complication occurred, just before the discharge. All patients were discharged in good clinical status and outpatient FOB was scheduled on the 30th postoperative day. Any further control was planned depending on patients’ clinical conditions and FOB outcomes.

### Statistical analysis

Data were analyzed using the software SPSS version 23.0 for Windows (Chicago, US). Continuous variables were expressed in terms of mean and standard deviation (SD) or median with range and interquartile range (IQR), while categorical variables were expressed in terms of frequency. Two-tailed Pearson’s Chi-square test was used for intergroup comparison of categorical variables while the Student’s *t*-test and one-way ANOVA test were used for continuous variables.

## Results

### Preoperative features

From May 2020 to October 2021, 15 patients underwent surgical tracheal resection followed by end-to-end anastomosis in our Center. There were 9 males (60%) and 6 females (40%), with mean age: 57.4 ± 13.21 years (range: 24–71 years). All patients suffered from benign tracheal stenosis due to prolonged invasive mechanical ventilation (14 patients, 93.3%) or previous laryngeal surgery for cancer (1 patient, 6.7%). Nine patients (60%) experienced previous SARS-CoV2 pneumonia that required OTI and/or tracheostomy. Mean OTI duration was 9.0 ± 5.1 days, while the average time of tracheostomy maintenance was 41.5 ± 50.2 days. Patients’ preoperative clinical and demographic features are reported in Table 1.

**Table 1:** Demographic and clinical preoperative features of patients operated on from May 2020 and October 2021 and benign tracheal stenosis’ features

Characteristic	Description
Sex (M/F; %)	9/6; 60%/40%
Mean age in years (range, SD)	57.4 (24–71; ± 13.2)
Previous SARS-CoV2 pneumonia (*n*, %)	9 (60%)
Major comorbidities (*n*, %)	Hypertension: 3 (27.3%)
	Cardiac arrhythmia: 3 (27.3%)
	COPD: 2 (18.2%)
	OSAS: 1 (9.1%)
	DM: 2 (18.2%)
	Multidistrict vasculopathy: 1 (9.1%)
	Myasthenia gravis: 1 (9.1%)
	Obesity: 2 (18.2%)
	Previous cancer: 3 (27.3%)
Mean BMI (range, SD)	28.3 (20–39; ± 4.9)
ECOG score (*n*, %)	1: 5 (33.3%)
	2: 3 (20.0%)
	3: 4 (26.7%)
	4: 3 (20.0%)
Mean OTI duration (days ± SD)	9.0 ± 5.1
Mean tracheostomy maintenance time (days ± SD)	41.5 ± 50.2
Tracheal stenosis etiology (*n*, %)	Prolonged IMV: 14 (93.3%)
	Previous tracheostomy: 12 (80%)
	Previous laryngeal surgery: 1 (6.7%)
Previous endoscopic treatment (*n*, %)	Laser therapy: 7 (46.7%)
	Dumon stent: 2 (13.3%)
	Montgomery T-tube: 1 (6.7%)
Mean distance between vocal cords and proximal margin of stenotic tract in mm (range, SD)	28.7 (15–85; ± 17.4)
Mean longitudinal extension of stenotic tract in mm (range, SD)	19.5 (10–30; ± 6.1)
Mean residual tracheal lumen % (range, SD)	35 (10–60; ± 18.1)
Complex stenosis (*n*, %)	12 (80%)

*N* number of patients, *SD* standard deviation, *ECOG* Eastern Cooperative Oncology Group, *COPD* chronic obstructive pulmonary disease, *OSAS* obstructive sleep apnea syndrome, *OTI* orotracheal intubation, *DM* diabetes mellitus, *IMV* invasive mechanical ventilation

All patients had a preoperative endoscopic evaluation while nine of them (60%) underwent endoscopic treatment of the tracheal stenosis by rigid bronchoscopy, which consisted in Nd–Yag laser therapy (7 patients, 46.7%) and/or positioning of endotracheal Dumon stent (2 patients, 13.3%) or Montgomery T-tube (1 patient, 6.7%). These subjects were subsequently addressed to surgical treatment after a median time of 1 months (range: 2 weeks–15 months, IQR: 2–1) mainly because of stenosis recurrence after laser disobstruction, stent complications such as migration or poor tolerance, or stent removal failure. Surgery was performed after a median of 7 months (range: 2–21 months, IQR 12–4) after prolonged OTI or tracheotomy.

### Stenosis characteristics

**Fig. 3:**
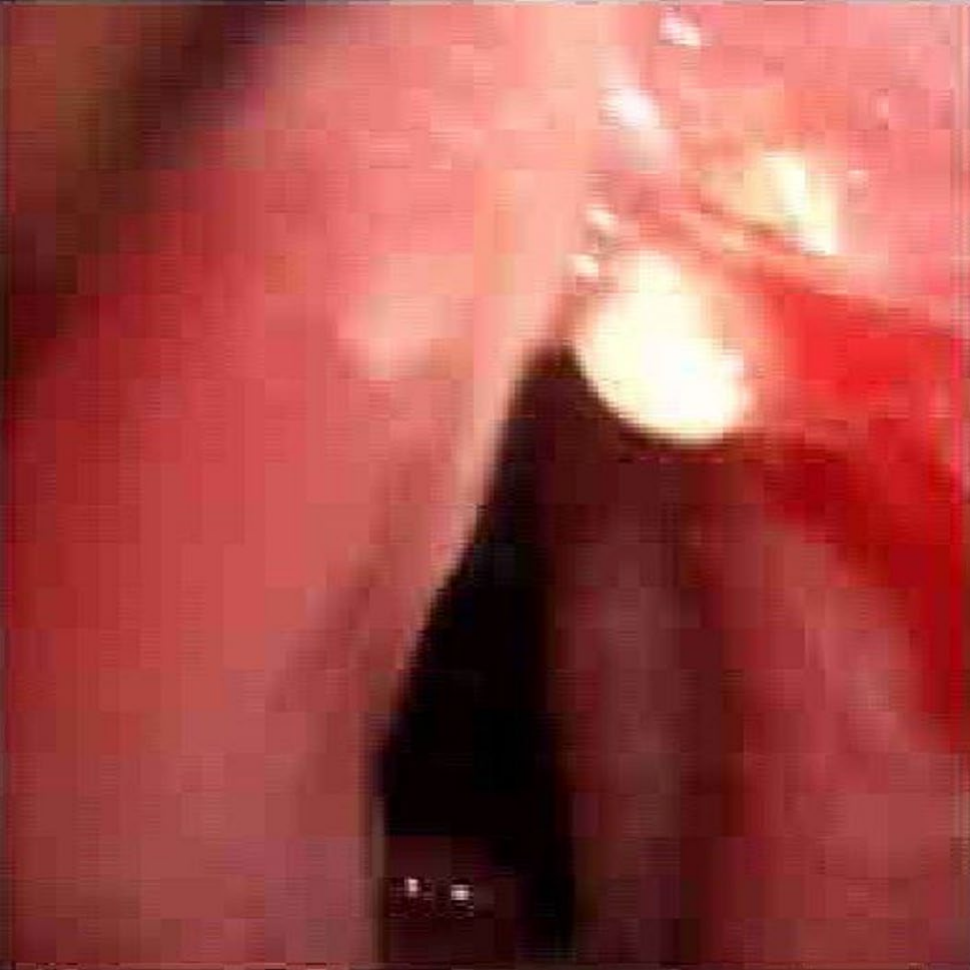
“Delta” morphology of tracheal lumen associated to broken cartilaginous rings

Tracheal stenosis features are described in Table 1. The mean distance between the proximal margin of stenosis and the rima glottis was 28.7 ± 17.4 mm (range: 15–85 mm) and the mean longitudinal extension was 19.5 ± 6.1 mm (range: 10–30 mm). The mean residual tracheal lumen was 35 ± 18% (range: 10–60%). Twelve patients (80%) suffered from complex tracheal stenosis, characterized by 1 or more cartilaginous rings rupture (Fig. 3), dynamic stenosis or length greater than 10 mm.

### Operative and pathologic details

Four patients (26.7%) had tracheostomy cannula in place at the time of surgery. One patient (6.7%) presented ruptured cricoid ring and underwent laryngo-tracheal resection and subsequent laryngo-tracheal end-to-end anastomosis, but no laryngeal release maneuvers were required because of the small longitudinal extension of the stenotic tract.

Mean operative time was 165 ± 28 min (range: 110–210 min) and mean length of tracheal specimen was 23.9 ± 6.5 mm (range: 15–38 mm). Pathology revealed mostly acute and chronic flogosis with lymphoplasmacellular infiltration, squamous metaplasia of perilesional epithelium and granulation tissue. In the specimens of five patients who experienced SARS-CoV2 infection (55.5%), pathologists observed lymphomonocytic perivascular infiltration, multiple necrosis spots together with microhemorrhages, small vessels vasculitis and vascular proliferation, not usually found in tracheal specimens before SARS-CoV2 pandemic.

### Postoperative course

Mean follow-up time was 18.9 ± 6.23 months. Median hospital stay was 10 days (range: 8–60 days, IQR: 23–8). Postoperative morbidity (within 30 days from surgery) rate was 53.3% (8 cases) and comprehended: three cases of unilateral vocal cord paresis (20%), one case of bleeding (6.7%) that required reintervention, one case of ab-ingestis pneumonia (6.7%), one case of wound infection (6.7%) and two cases of anastomosis dehiscence (13.3%), one of which later resulted in chronic tracheal stenosis. Morbidity occurred after a mean of 3.33 ± 2.18 days from surgery (range: 1–7). Infectious complications were treated with wound toilette and intravenous antibiotics. The only death occurred (6.7%, in 35th postoperative day) was due to severe respiratory failure in the patient who developed ab-ingestis pneumonia (Table 2).

**Table 2:** Postoperative morbidity grading according to CTCAE (Common Terminology Criteria for Adverse Events) v. 5.0

Postoperative morbidity	Grade	Number of patients (%)
Unilateral recurrent laryngeal nerve injury	2	3 (20%)
Hemorrhage	4	1 (6.7%)
Wound infection	3	1 (6.7%)
Anastomotic dehiscence	3	2 (13.3%)
Ab ingestis pneumonia	5	1 (6.7%)
Stenosis recurrence	3	1 (6.7%)

### Comparison between “post-Covid-19” group and “non-Covid-19” group

As reported in Table 3, the preoperative features of the two groups of patients were comparable in terms of demographic and clinical features and tracheal stenosis’ characteristics, except for the incidence of previous tracheostomy: every subject in the post-Covid group underwent percutaneous tracheostomy in ICU, versus 50% of the non-Covid group (*p* = 0.044).

**Table 3:** Comparison of demographic and preoperative clinical features, tracheal stenosis characteristics and intra- and postoperative details between patients who have had Covid-19 (“post-Covid”) and patients who did not (non-Covid”)

Characteristic	Post-Covid (*n* = 9)	Non-Covid (*n* = 6)	*p* value
Male sex (*n*, %)	6 (66.7%)	3 (50%)	0.455
Age in years (mean ± SD)	57.1 ± 9.9	57.8 ± 18.2	0.159
Mean BMI (± SD)	30.1 ± 5.6	26.3 ± 3.2	0.134
ECOG score > 2 (*n*, %)	4 (44.5%)	3 (50%)	0.595
Previous tracheostomy (*n*, %)	9 (100%)	3 (50%)	0.044*
Mean OTI duration (days ± SD)	10.9 ± 1.6	6.8 ± 6.9	0.131
Mean tracheostomy maintenance time (days ± SD)	30.4 ± 37.9	54.3 ± 61.8	0.376
Previous endoscopic treatment (*n*, %)	4 (44.5%)	4 (66.7%)	0.378
Distance of stenosis from vocal cords in mm (mean ± SD)	25.0 ± 9.3	34.2 ± 25.4	0.121
Longitudinal stenosis extension in mm (mean ± SD)	20.0 ± 5.9	17.4 ± 5.6	0.407
Residual tracheal lumen % (mean ± SD)	40.0 ± 16.0	30.0 ± 20.8	0.313
Complex stenosis (*n*, %)	8 (88.9%)	4 (66.7%)	0.446
Operative time in minutes (mean ± SD)	165.0 ± 34.0	165.0 ± 21.0	0.144
Length of resected specimen in mm (mean ± SD)	24.2 ± 7.2	23.3 ± 6.0	0.969
Length of postoperative stay in days (mean ± SD)	13.5 ± 7.6	20.0 ± 18.7	0.382
Postoperative morbidity rate (%)			
Acute (≤ 5th POD)	33.3%	33.4%	0.706
Late (> 5th POD)	22.2%	16.7%	0.659
Postoperative mortality rate (%)	0%	16.7%	0.467

*N* number of patients, *SD* standard deviation, *ECOG* Eastern Cooperative Oncology Group, *POD* postoperative day

*Statistically significant

The two groups were also similar in terms of operative times and postoperative course. More in details, interventions lasted for a mean of 165 ± 34 min in the post-Covid-19 group and 165 ± 21 min in the non-Covid-19 group (*p* = 0.144). Mean length of resected specimens was similar in the two groups (24.2 ± 7.2 mm vs. 23.3 ± 6.0 mm, respectively, *p* = 0.969). Patients who have had Covid-19 were discharged after a mean of 13.5 ± 7.6 days, while the other patients stayed in hospital for a mean of 20 ± 18.7 days (*p* = 0.382). Postoperative morbidity rates were similar in both groups; conversely, only post-Covid-19 patients presented anastomosis-related complications, already described.

## Discussion and conclusions

Tracheal stenosis is the most common long-term complication of acute airway injury whose management may be challenging, since it always requires a multidisciplinary approach and, often, a complex surgical treatment.

Since the first months of 2020, when SARS-CoV2 disease was designated a pandemic by the World Health Organization, a remarkable series of Covid-19 survivors are becoming an unprecedented object of study as regards upper airways physiopathology.

The novel Coronavirus pandemic has determined a significant increase in the proportion of critically ill patients needing IMV [11]: in fact, about 20% of Covid-19 patients develop a severe and critical disease and needs to be admitted in the ICU in 18–30% of cases, due to respiratory failure [12]. Moreover, up to 80–90% of those patients require intubation and, often, tracheostomy [13].

In this scenario, the role of surgery in the treatment of post-IMV (laryngo)tracheal stenosis, whose incidence has drastically grown, needs to be defined.

To date, the literature provides scarce information about the treatment of benign tracheal stenosis in patients who underwent IMV for SARS-CoV2-related respiratory failure: the few experiences reported are mostly case reports or small case series regarding subjects treated endoscopically or surgically. Even if the described results seem satisfying, it is not possible to draw any conclusion about the effectiveness of these approaches [14–18].

Our surgical Department, comprehensive of an Endoscopic Division, senior surgeons experienced in tracheal reconstruction and a dedicated ICU, has long been a reference point for patients suffering from (laryngo)tracheal stenosis. During the biennium 2020–2021, despite the reduction of surgical activity due to ICU congestion that characterized SARS-CoV2 pandemic, many subjects affected by airways stenosis came to our attention. Since nearly every hospital has been called to take charge of Covid-19 patients needing intensive cares, we speculate that post-IMV complications increasing rate may be somewhat related to the low experience in airways management and to the growing physical and emotional stress of many health practitioners who were not used to deal with it, besides to the specific Covid-19-related risk factors for tracheal damage [9] and the increased number of subjects with acute respiratory failure needing IMV.

Tracheal resection and reconstruction surgery is already known to be challenging and many precautions need to be observed to achieve satisfying results: the residual trachea should not be extensively dissected to avoid devascularization, suture must guarantee an adequate intraluminal caliber, and anastomosis protection with a muscular flap is sometimes required to avoid fistulas [19].

Our results, considering the small size of the study cohort, are encouraging, whereas patients with a history of SARS-CoV2 pneumonia seemed to have similar intra- and postoperative course than patients who never had Covid-19. Morbidity rates are about 50% in the whole cohort, with a prevalence of non-life-threatening events, while major morbidity that led to death occurred in only one patient (6.7%) who never encountered SARS-CoV2. Similar complications as those reported in this cohort are described also by Wright and colleagues in 2004 on a very large series of 901 patients [20]. In the literature, variable morbidity rates after tracheal surgical resection and reconstruction are reported, ranging from 5 to 45%. Bibas et al. found out a 44.6% morbidity rate on 94 patients (21%: anastomotic complications, 23.6%: non-anastomotic complications) [21]. In the same year, also Piazza and his team performed a retrospective study on 137 patients who underwent tracheal surgery for both benign and neoplastic stenosis, reporting a complications rate of 36% in neoplastic patients and 46% in patients operated for benign lesions [22].

Preoperative stenosis features were also comparable between the two groups, except for the number of tracheostomized patients, which was significantly higher in the post-Covid-19 group. The pandemic period has been characterized by a higher rate of tracheostomies than the pre-pandemic ones: in fact, some studies demonstrated that early (less than 10 days after OT intubation) tracheostomy favors Covid-19 patients by allowing sedation reduction, early rehabilitation and nutrition, and reducing laryngeal nerves injury [23].

Noticeably, the pathological report described, in the tracheal specimens of 55% of the “post-Covid” patients, signs of small vessel vasculitis, necrosis spots, vascular proliferation and microhemorrhage that were never seen before SARS-CoV2 pandemic [24]. These findings may be related to Covid-19-associated vasculopathy [25], even if poor evidence is available to date.

Only two “post-Covid” patients presented anastomosis-related complications. One of these two patients developed subsequently a tracheal stenosis recurrence; hence, he underwent endotracheal Dumon stent positioning. He was affected by diabetes mellitus, Myasthenia Gravis, and was under chronic steroid therapy; these comorbidities, together with the extension of the stenotic portion which required a tracheal resection of about 4 cm, may have played a role in the development of anastomosis dehiscence [26]. The other patient was still recovering from a large ischemic cerebral injury and, in this case, we may hypothesize that she had issues in avoiding cervical hyperextension, despite the presence of the chest-to-chin stitch. However, we do not have sufficient data to relate these complications to the previous SARS-CoV2 infection.

To conclude, although IMV is certainly recognized as a life-saving procedure for patients affected by respiratory failure, it has been demonstrated to result in injuries of the subglottic larynx and trachea, especially in patients affected by Covid-19 disease. Despite the progress of intensive care management concerning mechanical ventilation devices, post-IMV tracheal stenosis has shown to be a challenging issue. SARS-CoV2 pandemic made us face a major increase of post-IMV life-threatening airways complications, for which surgery is often the chosen curative treatment. Our data showed that tracheal surgery performed in patients who had Covid-19, even during such a healthcare system crisis period as the SARS-CoV2 pandemic, is as feasible and safe as in patients who never had SARS-CoV2 infection, if conducted in referral Centers by experienced and multidisciplinary equips.

## Data Availability

The data that support the findings of this study are available from the corresponding author, D.B., upon reasonable request.
